# Impact of a novel at-home, scope handle–based game controller on novice endoscopists

**DOI:** 10.1016/j.igie.2025.12.011

**Published:** 2026-01-10

**Authors:** Hadi Khaled Abou Zeid, Yara Salameh, John League, Benjamin Lange, Erik Clemens, Elizabeth Rajan, Nayantara Coelho-Prabhu, Cadman L. Leggett, Andrew C. Storm

**Affiliations:** 1 Division of Gastroenterology and Hepatology, Mayo Clinic School of Medicine, Rochester, Minnesota, USA; 2 Division of Engineering, Mayo Clinic Foundation for Medical Education and Research, Rochester, Minnesota, USA

## Abstract

**Background and Aims:**

Endoscopy training is an essential component of gastroenterology fellowship programs. Skill assessment has transitioned from a volume-based model to a competency-based framework. However, challenges such as limited supervising faculty availability, lack of standardized evaluations, and patient safety concerns hinder training. Simulation-based training has shown promise in enhancing endoscopic skills, but its adoption is restricted by cost and other practical constraints. To address these barriers, we developed ScopeQuest (Mayo Clinic, Rochester, Minn, USA), a novel, affordable, at-home, gaming-based endoscopy training device aimed at improving psychomotor skills in endoscopy trainees. This study aims to assess the feasibility and impact of the handheld ScopeQuest on endoscope manipulation skills.

**Methods:**

A single-arm pretest–post-test pilot study was conducted with medical trainees with no prior endoscopy experience. Participants were trained with the ScopeQuest. Pretraining and post-training endoscopic skill assessments were conducted. Participant confidence, perceived skill improvement, and satisfaction were evaluated using post-training surveys.

**Results:**

Ten participants (mean [standard deviation]) age 26 [1.25] years; 50% female) trained for an average (standard deviation) of 1080.5 (346.3) seconds. Mean (standard deviation) task completion time improved from 448.7 (218.85) to 243.6 (92.65) seconds (*P* = .509). Subjective assessments indicated increased confidence. Perceived technical skill improvement was noted by 87.5% of participants. Overall satisfaction was reported by 62.5% of participants.

**Conclusions:**

An at-home, gaming-based, handheld endoscope simulator is a feasible adjunct to traditional endoscopy training. Although preliminary results suggest improvements in task efficiency and trainee confidence, larger multicenter studies are needed to confirm its effectiveness and assess skill retention and translation to real-life clinical skill development.

## Introduction

Endoscopy training is an integral part of a gastroenterology fellowship program, designed to prepare trainees to diagnose and manage various disorders of the gastrointestinal (GI) system. The structure and content of endoscopy training programs vary across institutions and practice settings; however, in general, training is through an apprenticeship model, beginning through observation of experienced endoscopists and moving ideally at an individualized pace, toward the development of cognitive and procedural proficiency in scope manipulation and maneuvers. Because training advances, a learner might progress from performing basic diagnostic procedures to more complex therapeutic interventions, ultimately reaching competency as defined by several societies and governing bodies.^1^

Endoscopy training has transitioned from a volume-based model, in which competency was measured by the number of procedures performed, to a competency-based framework that prioritizes systematic objective assessment of trainees' skills.^2^ Professional endoscopy organizations such as the American Society for Gastrointestinal Endoscopy recommend a minimal threshold of procedures before competency can be assessed. The Accreditation Council for Graduate Medical Education has implemented new competency-based training requirements, emphasizing systematic documentation of procedural experience and performance assessments. Upon completing the fellowship, program directors certify a trainee's competence based on technical skills and overall endoscopy experience.^1^

This shift in endoscopy training has been hindered by several challenges within training programs.^2^^,^^3^ One major challenge is that endoscopists, who serve as the primary educators in this field, are often burdened with extensive clinical responsibilities, limiting the time and attention dedicated to training.^3^ In addition, many endoscopists lack formal training in educational methodologies, which can lead to poor training quality.^2^^,^^3^ Another key limitation is the absence of structured evaluations because many programs rely on subjective feedback rather than objective metric assessments, making it difficult for trainees to identify areas needing improvement and to track their progress over time.^2^ Furthermore, the lack of standardized educational frameworks across institutions, and sometimes even within the same institution, has been reported to create inconsistencies in learning experiences, causing confusion among trainees because they navigate different instructions.^2^^,^^3^ Finally, patient safety remains a critical concern, particularly in the early phases of training.^4^

Simulation-based training (SBT) in endoscopy has emerged as an essential component of modern medical procedure education because of its ability to enhance endoscopic skills in a low-risk setting.^4^, ^5^, ^6^ However, despite evidence supporting the effectiveness of SBT in enhancing technical proficiency, widespread adoption of SBT in endoscopy training remains limited because of logistical and financial constraints.^5^^,^^7^ In response to the challenges, and to overcome the constraints that prevent trainees from using SBT, we developed an internally manufactured and not yet commercially available pilot device called ScopeQuest (Mayo Clinic, Rochester, Minn, USA), a novel, inexpensive, at-home, gaming-based endoscopy training device designed to enhance the psychomotor skills of novice endoscopists. This pilot study aimed to assess the feasibility and impact of this device on endoscopy skills.

## Methods

In this pilot study, the impact of the ScopeQuest training in enhancing endoscopic manipulation skills was assessed among novice endoscopists. The study used a single-arm pretest–post-test design. Eligible participants included medical trainees without previous experience in operating a flexible endoscope or SBT. Participants were recruited via e-mail and in-person outreach at a single institution.^8^

Participants received a 10-minute orientation on the device and game set-up. They next completed 4 standardized tasks designed to simulate scope navigation. Each task required guiding the on-screen cursor using the up/down and left/right endoscope dials to identify and click on 10 virtual ulcers using the device's suction and water buttons (Video 1, available online at www.igiejournal.org).

All participants underwent an initial assessment of their endoscopic skills using GI Mentor (Surgical Science, Göteborg, Sweden), a commercially available and previously validated GI endoscopy simulator before beginning the intervention.^9^ The assessment involved completing the Endobasket task, where participants navigated an endoscope to retrieve 3 virtual basketballs and place them in a basket, with total time, in seconds, recorded. After the intervention, a second identical assessment was performed using GI Mentor to compare the time required to perform the same pretraining task. Post-training surveys were administered to determine participants’ attitude toward the handheld gaming-based endoscope simulator as well as their confidence and perceived skill improvement.

Completion times for the Endobasket task were automatically recorded by the GI Mentor software pretraining versus post-training for each participant. Survey responses were collected via an online questionnaire and summarized descriptively. All results were reported as mean (standard deviation [SD]). Paired *t* tests were used to compare pretraining and post-training task completion time. Statistical significance was set at *P* < .05, and analyses were performed using IBM SPSS Statistics version V.30 (Armonk, NY, USA).

## Device description

ScopeQuest is designed to replicate the functionality of a standard endoscope handle and is built on an inexpensive commercially available single-use scope handle platform (Ambu aScope Gastro, Ballerup, Denmark) that incorporates standard dual-control knobs that facilitate 2-dimensional navigation (left-right, up-down) and 2 push-button switches that mimic the water and suction functions of a real standard endoscope handle. An Arduino Micro Board (Arduino S.r.l., Lombardy, Italy) with custom firmware translates these actions from button clicks and potentiometer rotation into digital signals. The device connects to a computer via a standard universal serial bus (USB) cable. Once connected, the knobs control the movement of the mouse cursor within a custom-designed game that simulates the GI tract. The objective is to locate and interact with virtual ulcers using the water and suction buttons (Figs. 1 and 2). The estimated cost for the hardware components and modifications required to produce a single controller is $84 U.S. dollars and does not include the original cost of the Ambu scope control body itself. The software was developed internally at no cost by the engineering department.

**Figure 1:**
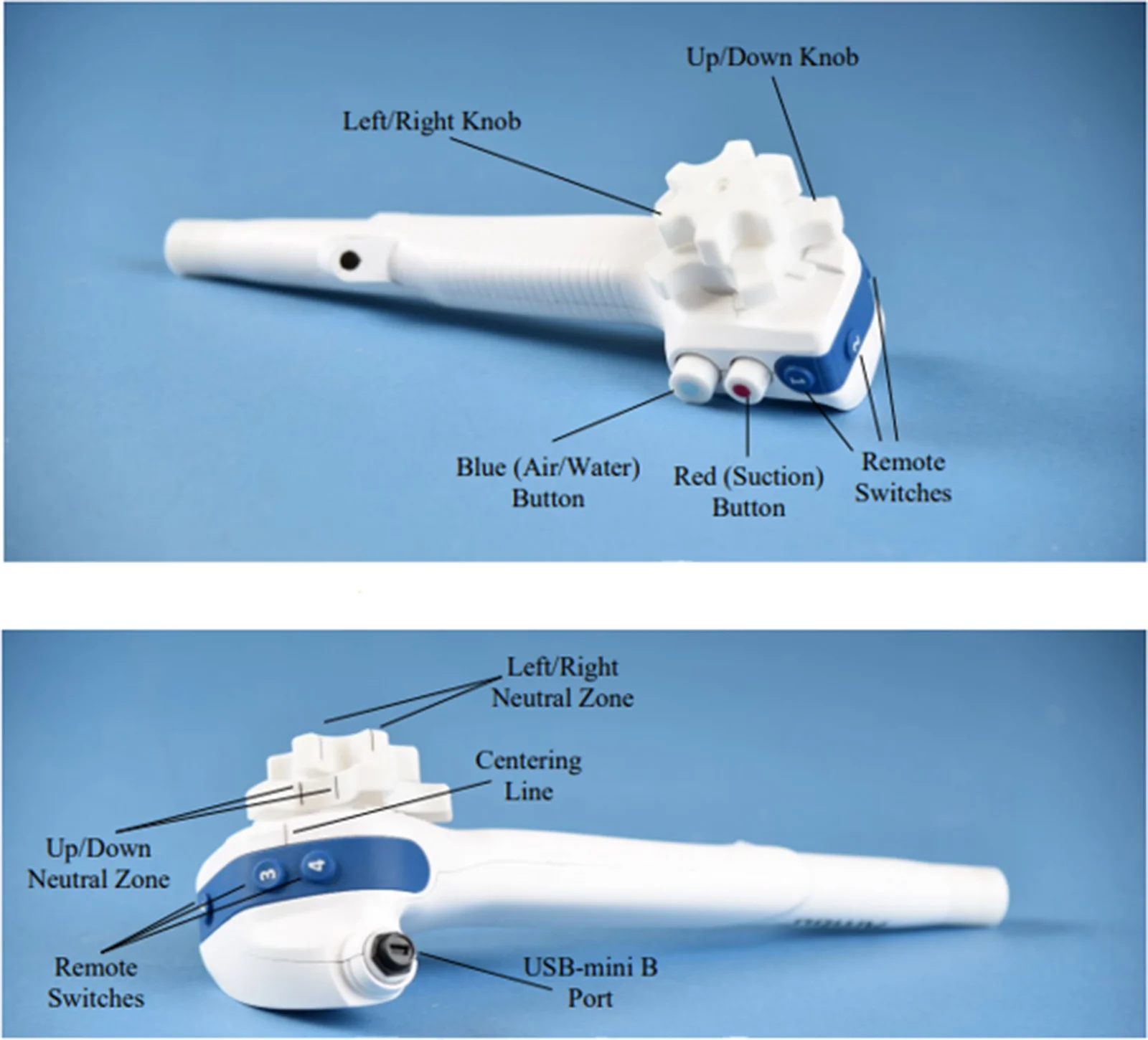
The ScopeQuest at-home endoscopy handle training device (Mayo Clinic, Rochester, Minn, USA). *USB*, Universal serial bus.

**Figure 2:**
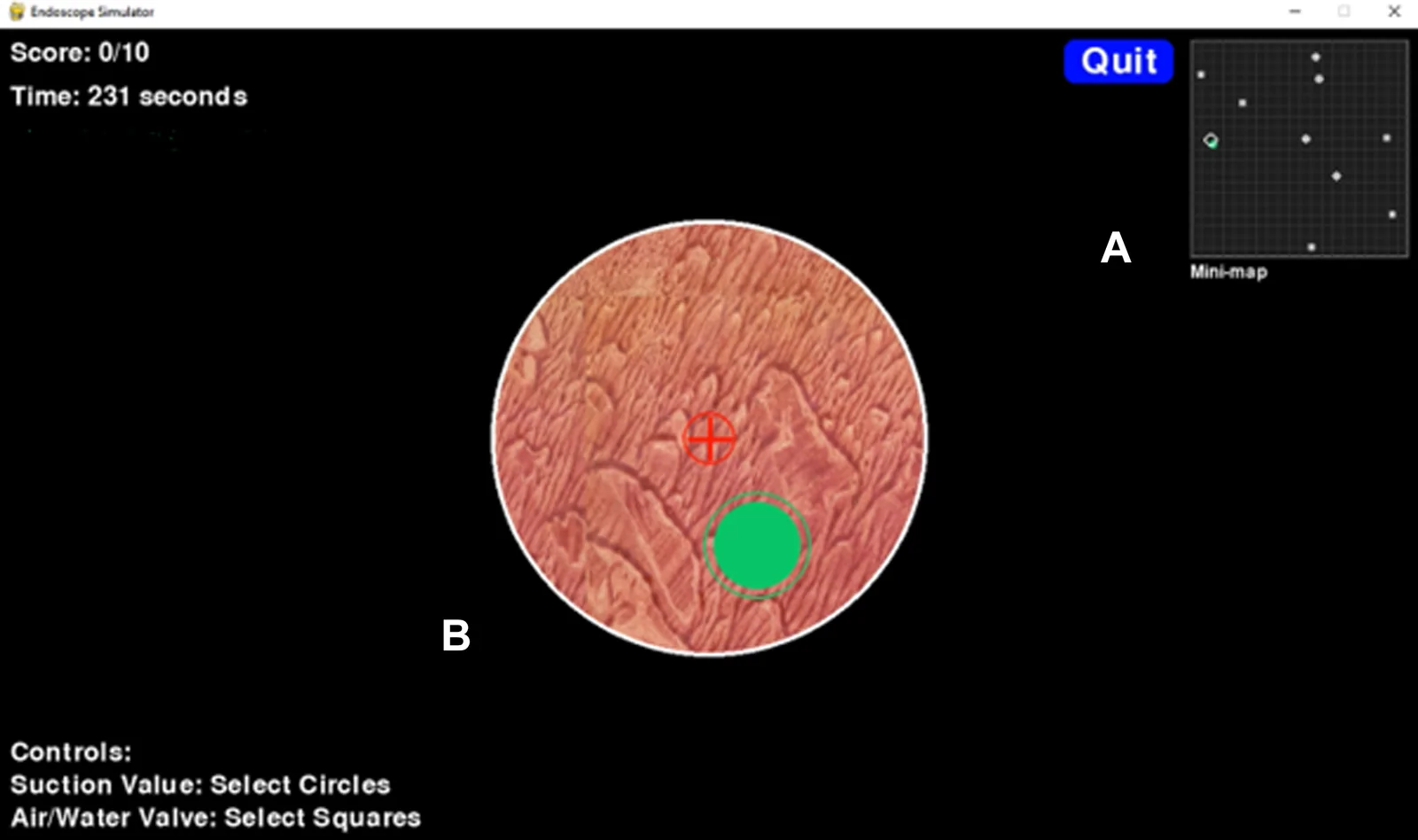
ScopeQuest (Mayo Clinic, Rochester, Minn, USA) software interface for ulcer localization and detection. The main circular view simulates the gastrointestinal mucosa, with the *green circle* representing an ulcer for training purposes. The *red crosshair* indicates the tip position controlled by the trainee. A mini-map (**A**) shows the relative positions of all targets. Control instructions are provided (**B**), where the suction button selects circles and the air/water valve selects squares.

## Results

Ten individuals (mean [SD] age 26 [1.25] years; 50% female), with no endoscopic experience, participated. Participants underwent practice sessions using the ScopeQuest for a mean (SD) duration of 1080.5 (346.3) seconds. All participants completed the prescribed 4 training tasks on the device without any reported technical issues or malfunctions. The mean (SD) time required to complete the standardized Endobasket task assessment on the GI Mentor system decreased from 448.7 (218.85) to 243.6 (92.65) seconds (*P* = .509) after training (Fig. 3).

**Figure 3:**
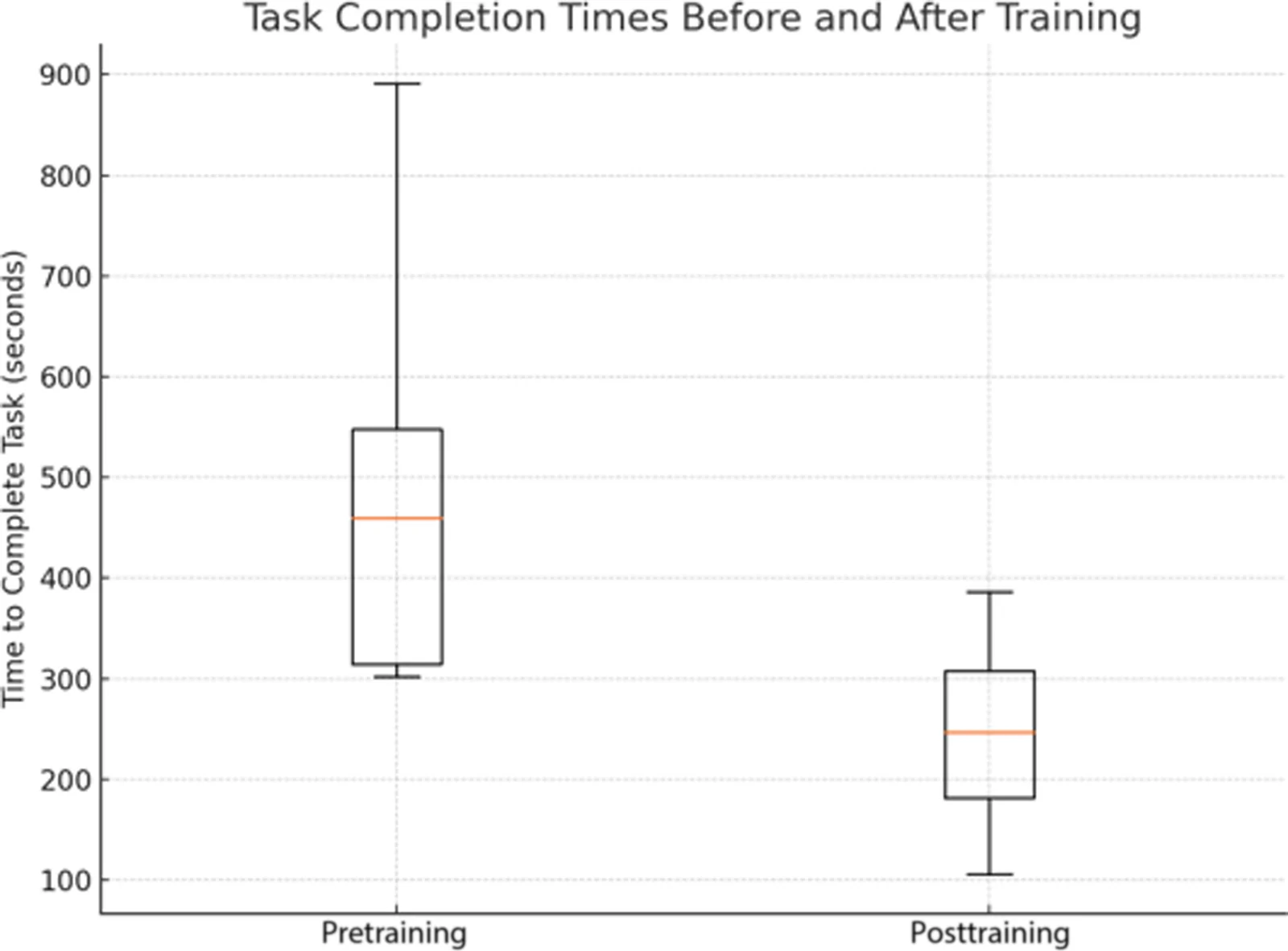
Boxplot comparing the time required to complete the Endobasket assessment before and after training with the ScopeQuest device (Mayo Clinic, Rochester, Minn, USA).

Subjectively, before using the handheld endoscope, 62.5% (5/8) of participants reported being “not confident” in performing endoscopic procedures, whereas 25% (2/8) were only “slightly confident” and 12.5% (1/8) were predominantly “confident.” After training with a handheld endoscope, confidence levels improved: 50% (4/8) of participants rated themselves as “fairly confident,” 25% (2/8) remained “slightly confident,” and another 25% (2/8) reported being “very confident.” Moreover, 87.5% (7/8) noted perceived improvements in their technical skills, and 12.5% (1/8) strongly disagreed. However, when assessing overall preparedness for performing endoscopic procedures, responses varied: 37.5% (3/8) agreed that they felt more prepared, 25% (2/8) remained neutral, 25% (2/8) strongly disagreed, and 12.5% (1/8) disagreed. In terms of engagement, the training experience was rated with 37.5% (3/8) considering it “excellent,” another 37.5% (3/8) rating it “very good,” and 25% (2/8) finding it “satisfactory.” As for satisfaction with the ScopeQuest training, 62.5% (5/8) of participants reported that they were “very satisfied,” 25% (2/8) reported being “satisfied,” and 12.5% (1/8) reported being neutral. Regarding the likelihood of recommending the ScopeQuest to colleagues, 75% (6/8) indicated that they were “very likely” to do so, whereas 25% (2/8) said that they were “likely.”

## Discussion

Our preliminary findings suggest that the ScopeQuest may help endoscopy trainees improve their endoscope manipulation skills, as evidenced by the reduction in mean (SD) time from 448.7 (218.85) to 243.6 (92.65) seconds in completing a task with a validated endoscopy simulator system, although this difference did not reach statistical significance because of the small sample size. Participants also reported increased confidence and positive attitudes toward the handheld gaming-based endoscope. These findings highlight the feasibility of the ScopeQuest as a potentially accessible and cost-effective educational at-home tool for early endoscopic training.

SBT in endoscopy had a positive impact on endoscopy training.^4^, ^5^, ^6^ One of the key advantages of SBT in endoscopy is the opportunity for trainees to develop psychomotor and cognitive skills through deliberate repetitive practice with clearly defined learning objectives.^4^^,^^6^ For instance, studies have shown that SBT accelerates skill acquisition in novice endoscopists and improves patient safety. Moreover, virtual reality (VR) simulators allow trainees to practice various endoscopic techniques while receiving immediate performance feedback, helping trainees identify key areas for improvement.^5^ Despite these benefits, widespread adoption of SBT in endoscopy remains limited because of financial and practical constraints. One significant obstacle is the high cost associated with VR simulators, which limits their accessibility in resource-limited programs.^5^^,^^7^ Although mechanical models are more cost-effective, they frequently lack the anatomical and tissue realism necessary for effective skill transfer to clinical practice. All these systems are also unlikely to be available to trainees before arriving at their training programs, making an at-home personal computer-based system attractive. In addition, although simulation is highly effective for early skill acquisition, there is limited evidence to support whether these skills can be translated into real-life endoscopic procedures.^5^ Our findings build upon the established benefits of SBT and offer an at-home simulation solution that addresses some of the financial and logistical barriers facing SBT implementation.

A unique advantage of our device is its at-home accessibility, which allows trainees to practice independently at their convenience without the constraints of scheduled on-site sessions. With USB compatibility, it can be used on any standard computer, enabling flexible and self-directed learning. Although the final pricing of a future commercially available ScopeQuest device will depend on any additional modifications or refinements, it will remain substantially more affordable than the commercially available VR simulators, which range from $62,087 to $136,591 U.S. dollars.^10^ Its cost ensures that training programs can equip trainees with early access to endoscopic skill development. This early exposure enables individuals to develop proficiency in endoscope maneuvering, thereby facilitating a smoother transition to more complex procedures during fellowship. Although the ScopeQuest does not include a full endoscope shaft, which may limit the development of scope handling, bimanual dexterity, and ergonomic skills, it still effectively promotes hand-eye coordination and tip control in the up-down and right-left directions. These fundamental skills represent a critical foundation for more advanced endoscopic tasks and serve as an important first step in the learning curve. Furthermore, the integration of gamification enhances engagement and motivation, providing an interactive and immersive learning experience. It also has the potential to improve training on a larger world scale. In fact, this inexpensive technology can be used in underserved resource-poor communities to enhance skills for all practitioners, including nongastroenterologists who provide care in these settings.

Although our preliminary results suggest a potential improvement in efficiency, the results did not reach statistical significance because of the small sample size and variability in individual performance. Because it is a single-arm study, we could not compare our findings with alternative simulators or standard practice. Moreover, the immediate post-training assessment may not capture long-term skill retention and may not reflect translation of these skills into real endoscopy. To address these limitations, larger multicenter studies are warranted.

Looking ahead, the device holds significant potential for expansion and integration with emerging technologies. Its adaptability allows seamless connectivity to various platforms, including VR systems, enabling trainees to engage in immersive, at-home simulations. Future developments could include a dedicated platform, where users can download customized training modules from one of several commercial software application platforms, anywhere in the world.

In conclusion, our pilot study suggests that the at-home, gaming-based handheld ScopeQuest training device presents a feasible approach to early endoscopic skill development. It is designed to complement available endoscopy training platforms in enhancing skill acquisition and accelerating trainees' learning curves. Further studies are warranted to understand its efficacy and define its potential within endoscopy training.

## Patient consent

Oral informed consent was obtained from all participants before their involvement in the study, according to the local Institutional Review Board's requirements.

## Disclosure

The following authors disclosed financial relationships: E. Rajan: Intellectual property with Medtronic, Ruhoff; consultant for Olympus; research support from Mirai Medical, CapsoVision. A. Storm: Research support from Apollo Endosurgery, Boston Scientific, Endogenex, Endo-TAGSS, Enterasense, EnVision, MGI Medical, OnePass, SofTac; consultant for Ambu, Boston Scientific, Cook, Intuitive, Medtronic, Micro-tech, Olympus. N. Coelho-Prabhu: Consultant for Iterative Health; speaker for Boston Scientific. All other authors disclosed no financial relationships. Ambu provided expired single-use gastroscopes to serve as the handle for our training device. Mayo Clinic provided internal funding for engineering the scope handle and gaming platform for training.

## Supplementary data

Video 1 Real-time demonstration of the gaming-based endoscopy training device, ScopeQuest.
